# Physical inactivity and its associated factors among pre-retirement government healthcare workers in Kuala Lumpur, Malaysia

**DOI:** 10.1186/s12889-024-19411-y

**Published:** 2024-07-18

**Authors:** Rahmat Dapari, Mohamad Rabani Abdul Wahab, Mohd ‘Ammar Ihsan Ahmad Zamzuri, Mohd Rohaizat Hassan, Nazri Che Dom, Syed Sharizman Syed Abdul Rahim

**Affiliations:** 1 Department of Community Health, Universiti Putra Malaysia, Serdang, 43400 Malaysia; 2 State Department of Health Negeri Sembilan, Negeri Sembilan, Seremban, 70300 Malaysia; 3 Department of Community Health, Universiti Kebangsaan Malaysia, Cheras, 56000 Malaysia; 4 Faculty of Health Sciences, Universiti Teknologi MARA, Bandar Puncak Alam, 42300 Malaysia; 5 Public Health Medicine Department, Faculty of Medicine and Health Sciences, Universiti Malaysia Sabah, Kota Kinabalu, 88400 Malaysia

**Keywords:** Physical inactivity, Healthy ageing, Government worker, Healthcare worker, Physical activity barrier, Workplace health promotion

## Abstract

**Introduction:**

The rising worldwide concern of Non-Communicable Diseases (NCD) is alarming as it is associated with 80% of annual global mortality. NCD threat is rising due to, among others, the increasing ageing population, thus putting the efforts to promote health ageing at the forefront of many countries’ health agenda. Physical activity has been recognised as one of the significant factors in the pursuit of healthy ageing. Nevertheless, approximately one third of individuals in Malaysia are physically inactive. The aim of this study is to determine the prevalence of physical inactivity and its associated factors among pre-retirement government healthcare workers.

**Methods:**

This cross-sectional study was conducted from May to June 2023 among pre-retirement government healthcare workers in Kuala Lumpur, Malaysia. The sample size required was 233 and proportionate random sampling was used to recruit potential respondents who answered self-administered online questionnaires. Global Physical Activity Questionnaire (GPAQ) was used to measure the level of physical activity and data analysis was performed using SPSS version 29.

**Results:**

A total of 214 complete responses were received from the 233 questionnaires distributed, giving a response rate of 91.8%. The prevalence of physical inactivity among pre-retirement healthcare workers was 39.7% as compare only 29.9% in general population. Significant predictors for physical inactivity included higher education levels (SPM, STPM, or certificate holders) (AOR = 13.4, 95% CI: 2.47–72.65), non-Malay ethinicity (AOR = 4.7, 95% CI: 1.23–18.38), personal barriers (AOR = 1.6, 95% CI:1.35–1.79), social barriers (AOR = 1.21, 95% CI: 1.06–1.39), and physical environment barriers (AOR = 1.468, 95% CI: 1.221–1.765).

**Conclusion:**

This study shows a worrying prevalence of physical inactivity among pre-retirement healthcare workers that is even higher than the general population in Malaysia. The findings highlight the importance of focusing the preventive strategies among non-Malay workers and those with lower education levels. It is also vital to address all the physical, social, and environmental barriers towards physical inactivity. By prioritising these factors, employers and stakeholders will be able to establish better workplace health promotion and address the issue of physical inactivity more efficiently.

## Introduction

According to the World Health Organisation (WHO), healthy ageing can be defined as the process of developing and maintaining functional abilities that enable wellbeing in older age [1]. The importance underscoring such action is growing with the shift in the global population structure whereby one in six individuals in the world will be above 60 years old by 2030 [1]. Similarly, Malaysia is expected to have an ageing population by 2034 with 15% of the total population above 60 years of age [2]. Due to the close association between the ageing population and increased risks of multiple chronic health issues such as diabetes, cardiovascular diseases, cerebrovascular disease, osteoporosis, obesity, cognitive problems, and vision issues, it becomes more imperative to address the issues pertaining to ageing population both nationally and globally [1, 3].

Workplace health promotion is defined as the cumulative efforts of workers, companies, and communities to improve the health and wellbeing of the working population [4]. With a high labour force participation rate of 70% in 2022, workplace health promotion is an ideal strategy to address various health issues of the working population in Malaysia [5]. For instance, the healthy ageing agenda can be disseminated among the pre-retirement working population as previous evidence showed that workplace programmes that target the pre-retirement population can cast a positive impact on healthy ageing [6, 7]. Generally speaking, pre-retirement workers include those in the last decade of employment before retirement [8, 9]. Among Malaysian government employees, the retirement age ranges between 55 and 60 years old, thus the pre-retirement workers refer to those between 45 and 60 years old.

Being physically active is a significant determinant of healthy ageing [10]. However, physical inactivity continues to be a problem in Malaysia which 29.9% reported themselves as physically inactive in the National and Health Morbidity Survey 2023 (NHMS). Furthermore, the prevalence of physical inactivity increases with age, with the highest prevalence being recorded among those above 75 years old. In addition, urban dwellers in cities such as Kuala Lumpur and government employees also reported a higher prevalence [11]. Between 2001 and 2016, the prevalence of physical inactivity rose by 5.0% from 32.0 to 37.0% in high-income countries [12]. Following the COVID-19 pandemic in 2020, many studies also highlighted an escalating trend of physical inactivity in multiple countries, including Iran (78.0%), Brazil (79.4%), and Malaysia (49.1%) [13–15].

The magnitude of physical inactivity and its complications can cast various negative impacts on the population. At the individual level, it increases the risk of non-communicable diseases (NCD) such as cardiovascular disease, breast cancers, fractures, falls, cognitive decline, Alzheimer’s Disease, depression, and functional limitations [16]. At a broader population level, it is estimated that physical inactivity costs the healthcare system up to USD 54 billion a year [17], thus highlighting the necessity to mitigate the problem.

To date, studies have shown a higher prevalence of physical inactivity among healthcare workers (HCWs) than the general population, ranging between 32.8% and 45.6% [18–20]. HCW is defined as personnel employed to work in health facilities, such as doctors, nurses, medical assistants, and laboratory technicians, as well as administrative staff such as clerks and accountants [21]. HCWs are expected to actively promote healthy lifestyles among patients, their families, and the broader community. Additionally, government-employed HCWs embody the government’s image in the health sector to the public. Thus, the aim of this study is to determine the prevalence of physical inactivity and its associated factors among pre-retirement government healthcare workers. The results of this study can be used to facilitate employers in developing effective intervention programmes that can enhance physical activity among HCWS.

## Methods

### Study design and study location

A cross-sectional study was conducted among HCWs in Kuala Lumpur. The estimated population of Kuala Lumpur is 1.75 million individuals [22]. The study was performed among HCWs in a government health department in Kuala Lumpur, i.e. the Kuala Lumpur State Health Department which encompasses the state health department headquarters, four district health offices, 24 health clinics, 22 community clinics, and a tertiary specialised hospital.

The inclusion criteria for this study were (i) workers between 45 and 60 years old, (ii) permanent staff employed by the Public Service Department, and (iii) serving the Kuala Lumpur State Health Department. The exclusion criteria were (i) workers who were on long leave due to sickness, study leave, confinements, or other reasons, (ii) workers who could not follow instructions to answer the online questionnaire, and (iii) workers with physical and/ or intellectual disabilities.

### Sampling frame and sample size

We estimated the required sample size for each potentially associated factor based on the number of pre-retirements HCWs working under the department using the two proportions formula. The highest sample size required for this study with 95% precision and 80% power, was estimated to be 194 based on various factors of occupational injury [23]. To account for a 20% dropout rate, the final sample size was inflated to 233. Based on updated list of health care workers in State Health Department as well as District Health Office, there were 424 eligible workers that full fill the inclusion criteria.

### Study instruments and data collection

Self-administered questionnaires in both English and Malay languages were distributed via Google Forms. The link of Google Forms was delivered to selected respondent via WhatsApp and email. The questionnaire was divided into seven sections. Section 1 consists of six items on the sociodemographic lifestyle factors. Section 2 consists of seven health-related items, including the presence of various NCDs, and the respondent’s self-reported weight and height for the calculation of Body Mass Index (BMI). Asian Classification of Weight Categories (Ministry of Health Malaysia, 2004) were used for BMI classification. Section 3 contains 15 questions from the Global Physical Activity Questionnaire (GPAQ) to measure the level of physical activity of the respondents. GPAQ has been recognised internationally as a valid instrument for different environments and cultures [24]. The Malay-translated version of GPAQ has been validated by Soo et al. [25]. In this study, physical activity levels were classified as physically inactive if the measurement < 600 Metabolic Equivalent of Task (MET-min/week) based on the recommendation [25].

Section 4 consists of four items on occupational factors, including job title and grade, current workplace, and work schedule. Section 5 contains 22 items that evaluated barriers towards physical activity, with 13 questions on personal barrier factors, five questions on social barrier factors, and four questions on environment barrier factors. All questions were assessed on a five-point Likert Scale, with higher scores indicating a higher influence of the factor towards physical activity. All questions in this section were adapted from Zakariah et al. [20]. The Cronbach alpha value was 0.92, indicating a good reliability of the questionnaire.

Section 6 consists of one question on lifestyle factors, i.e. smoking status, whether they are still smoking, have quit for the past six months, or never smoked before. Section 7 consists of two items adapted from Knox et al. [26] on knowledge of physical activity guidelines. The respondents must answer an open-ended question on the minimum recommended duration of moderate and vigorous physical activity recommended by health professionals. Each correct answer was given one point while zero point was given for incorrect answer. The total knowledge score was the sum of the scores for both items. Individuals receiving two points were regarded as having good knowledge, one point as having fair knowledge, and zero points as poor knowledge. A reliability test was performed for this section. The Cohen’s Kappa score was 0.66, indicating a moderate agreement between both scores and the knowledge level [27].

For data collection, a proportional random sampling method was used for all the above-mentioned centres under the Kuala Lumpur State Health Department. The sample population from all six responsibility centres was randomly sampled using an online random generator based on the name list of HCWs provided by the responsibility centres. The sampled respondents were then given a link to answer the questionnaire on Google Forms through a liaison officer from each responsibility centre. They were asked to read the information sheet that outlined the study details and their eligibility to participate before agreeing to participate in the study. Liaison officer will reconfirm the eligibility of the respondent prior the data collection. The data collection took place from May until June 2023.

### Data analysis

Data analysis was carried out using IBM SPSS Statistics for Windows, Version 29.0.2.0 Armonk, NY: IBM Corp. Numerical data were presented using mean and standard deviation for normally distributed data while median and interquartile range were used to present non-normally distributed data. For categorical data, frequency and percentage were used to describe the respondents’ characteristics. All hypothesis testing in this study used a two-directional test with the significance level (α) set at 0.05. The Chi-square test was used to determine the association between the dependent variable and all the categorical independent variables. For continuous variables, the association was analysed using Simple Logistic Regression (SLR). The predictors for physical inactivity were determined by using Multiple Logistic Regression (MLR) analysis. Variables with p-value < 0.25 were inserted into the model and those with a significance level of < 0.05 were regarded as significant predictors of physical inactivity.

## Results

A total of 214 completed responses were received from the 233 questionnaires distributed via Google Form (Response rate: 91.8%). Age and monthly household income were not normally distributed whereas other numerical data including BMI, personal barrier factor, social barrier factor, and environment barrier factor were all normally distributed.

The prevalence of physical inactivity among pre-retirement HCWs was as high as 39.7% (Table 1). The majority of the respondents were below 49 years old (61.7%), females (74.3%), and Malay (83.6%). Most of them were married (82.2%), with a monthly household income of less than RM 9,000 per month (42.1%), and with a degree or post-graduate as the highest education level (49.5%).

**Table 1:** Prevalence of physical inactivity and sociodemographic characteristics of the respondents (*n* = 214)

Variables	*n*	%
**Physical activity status**		
Physically Inactive	85	39.7
**Physically Active**	129	60.3
**Age (years)**		
45–59	132	61.7
50–54	55	25.7
55–60	27	12.6
**Gender**		
Male	55	25.7
Female	159	74.3
**Ethnicity**		
Malay	179	83.6
Chinese	4	1.9
Indian	23	10.7
Others	8	3.7
**Marital Status**		
Single	18	8.4
Married	176	82.2
Widowed/ Divorced	20	9.3
**Education Level**		
Degree/Post-Graduate	106	49.5
Diploma	86	40.2
SPM/STPM/Certificate	22	10.3
**Monthly Income (RM)**		
< 9,000	102	47.7
9,000–15,999	79	36.9
16,000 and above	33	15.4

In this study, 43% of respondents reported having at least one health issue, with 14% with hypertension, 8.4% with diabetes, 9.3% with asthma, and others (Table 2). Most of them were overweight based on the BMI classification (40.7%). In terms of occupation, about half were support staff (54.7%) while 54.3% worked in clinics. The majority of them followed an office-hour work schedule. Besides that, 90.7% of the respondents were non-smokers. In terms of knowledge, 70.1% had poor knowledge of the recommended physical activity guidelines.

**Table 2:** Health status, occupational factors, smoking status, and knowledge of respondents (*n* = 214)

Variables	*n*	%
**Disease Presence**		
Overall		
No	122	57.0
Yes	92	43.0
**Hypertension**		
No	184	86.0
Yes	30	14.0
**Diabetes**		
No	196	91.6
Yes	18	8.4
**Asthma**		
No	194	90.7
Yes	20	9.3
**Musculoskeletal Problem**		
No	199	93.0
Yes	15	7.0
**Hypercholesterolaemia**		
No	168	78.5
Yes	46	21.5
**Other Diseases**		
No	202	94.4
Yes	12	5.6
**BMI**		
Underweight (≤ 18.5 kg/m2)	13	6.1
Normal Weight (18.5–22.9 kg/m2)	44	20.6
Overweight (23–27.4 kg/m2)	87	40.7
Obese (≥ 27.5 kg/m2)	70	32.7
**Occupation Type**		
Support Workers	117	54.7
Management and Professional	97	45.3
**Work Place**		
Hospital	52	24.3
Clinic	97	45.3
Health Office	65	30.4
**Work Schedule**		
Shift Schedule	22	10.3
Office Hours	192	89.7
**Smoking Status**		
Smoker	9	4.2
Non-smoker	194	90.7
Ex-Smoker	11	5.1
**Knowledge**		
Poor	150	70.1
Fair	36	16.8
Good	28	13.1

The mean score for personal, social, and physical environmental barrier factors as 30.3 ± 7.11, 13.3 ± 4.14, and 10.3 ± 3.0 respectively (Table 3). Under each category, the factors most strongly agreed by the respondents as a barrier were the lack of self-discipline (20.6%) for personal barriers, no free or spare time (16.8%) under social barriers, and hot weather and rain under personal environment barriers (19.2%).

**Table 3:** Distribution of barriers to physical activity among respondents (*n* = 214)

Variables	SD		D		*N*		A		SA		Mean ± S.D
	*n*	(%)	*n*	(%)	*n*	(%)	*n*	(%)	*n*	(%)	
**Personal Barriers**											30.3 **±** 7.11
Tired	25	11.7	60	28.0	69	32.2	46	21.5	14	6.5	
Lazy	27	12.6	60	28.0	77	36.0	41	19.2	9	4.2	
Self-ashamed	120	56.1	65	30.4	20	9.3	6	2.8	3	1.4	
Don’t know the correct way	67	31.3	70	32.7	52	24.3	21	9.8	4	1.9	
Activities are active enough	42	19.6	61	28.5	59	27.6	41	19.2	11	5.1	
Afraid of Injury	69	32.2	64	29.9	47	22.0	29	13.6	5	2.3	
Medical condition	111	51.9	60	28.0	26	12.1	11	5.1	6	2.8	
Cause muscle and joint pain	34	15.9	68	31.8	55	25.7	43	20.1	14	6.5	
Body shape	98	45.8	79	36.9	24	11.2	10	4.7	3	1.4	
Fasting	55	25.7	62	29.0	54	25.2	31	14.5	12	5.6	
Inconvenient	61	28.5	75	35.0)	51	23.8	22	10.3	5	2.3	
Boring	96	44.9	73	34.1	32	15.0	8	3.7	5	2.3	
Lacking self-discipline	27	12.6	39	18.2	55	25.7	49	22.9	44	20.6	
**Social Barriers**											13.3 ± 4.14
No encouragement from family/ friends	94	43.9	50	23.4	40	18.7	23	10.7	7	3.3	
No free time	36	16.8	35	16.4	56	26.2	51	23.8	36	16.8	
No company	35	16.4	54	25.2	61	28.5	42	19.6	22	10.3	
Interrupted work or daily chores	41	19.2	52	24.3	59	27.6	40	18.7	22	10.3	
Social or family activity interruption	51	23.8	56	26.2	55	25.7	36	16.8	16	7.5	
**Physical Environment Barriers**											10.3 ± 3.0
Expensive and need to spend money	60	28	91	42.5	40	18.7	20	9.3	3	1.4	
Weather issues	14	6.5	32	15.0	53	24.8	74	34.6	41	19.2	
Facilities unavailable	43	20.1	73	34.1	62	29.0	28	13.1	8	3.7	
Facilities too far	53	24.8	77	36.0	59	27.6	18	8.4	7	3.3	

*SD = strongly disagree*,* D = disagree*,* N = neutral*,* A = agree*,* SA = strongly agree*

S.D = standard deviation

All question items are positive items towards each factor

The age group with the highest prevalence of physical inactivity was those of 55 years to 60 years old (59.3%), female (42.8%), non-Malay (54.3%), married (40.3%), with monthly household incomes of less than RM9,000, and had higher education levels (degree and post-graduate) (48.1%) as shown in Table 4. Table 5 shows that respondents with at least one NCD reported a higher prevalence of physical inactivity (41.3%) compared to those without (38.5%). The highest prevalence of physical inactivity was among those with asthma (60%) Musculoskeletal problems (46.7%), and diabetes (44.4%). In addition, the prevalence of physical inactivity was higher in the obese group (44.3%), among management and professional groups of workers (50.5%), those working in health offices (49.2%), and those following office hours work schedules (40.6%).

**Table 4:** Association between sociodemographic characteristics and physical inactivity among respondents (*n* = 214)

Variables	Physically Inactive *n* (%)	Physically Active *n* (%)	χ2	df	*P*-value
**Age (years)** 45–49	40 (30.3)	92 (69.7)	13.081	2	0.001*
50–54	29 (52.7)	26 (47.3)			
55–60	16 (59.3)	11 (40.7)			
**Gender** Male	17 (30.9)	38 (69.1)	2.400	1	0.121
Female	68 (42.8)	91 (57.2)			
**Ethnicity** Malay	66 (36.9)	113 (63.1)	3.708	1	0.054
Non-Malay	19 (54.3)	16 (45.7)			
**Marital Status** Married	71 (40.3)	105 (59.7)	0.160	1	0.689
Non-Married	14 (36.8)	24 (63.2)			
**Education** Degree/ Postgraduate	51 (48.1)	55 (51.9)	14.976	2	< 0.001*
Diploma	21 (24.4)	65 (75.6)			
SPM/STPM/Certificate	13 (59.1)	9 (40.9)			
**Monthly Income** **(RM)**					
≤ 9,000	31 (30.4)	71 (69.6)	8.794	2	0.012*
9,001–15,999	35 (44.3)	44 (55.7)			
16,000 and above	19 (57.6)	14 (42.4)			

* significant at p-value < 0.05

**Table 5:** Association between health status, occupational factors, smoking status and knowledge with physical inactivity among respondents (*n* = 214)

Variables	Physically Inactive *n* (%)	Physically active *n* (%)	χ2	df	*p*-value
**Disease Presence (Overall)** No	47 (38.5)	75 (61.5)	0.169	1	0.681
Yes	38 (41.3)	54 (58.7)			
**Diabetes Mellitus** No	77 (39.3)	119 (60.7)	0.183	1	0.669
Yes	8 (44.4)	10 (55.6)			
**Hypertension** No	73 (39.7)	111 (60.3)	0.001	1	0.973
Yes	12 (40.0)	18 (60.0)			
**Asthma** No	73 (37.6)	121 (62.4)	3.790	1	0.052
Yes	12 (60.0)	8 (40.0)			
**Musculoskeletal Problem** No	78 (39.2)	121 (60.8)	0.325	1	0.569
Yes	7 (46.7)	8 (53.3)			
**Hypercholesterolaemia** No	65 (38.7)	103 (61.3)	0.346	1	0.557
Yes	20 (43.5)	26 (56.5)			
**Other Disease** No	80 (39.6)	122 (60.4)	0.020	1	0.887
Yes	5 (41.7)	7 (58.3)			
**BMI Status** Non-Obese	54 (37.5)	90 (62.5)	0.906	1	0.341
Obese	31 (44.3)	39 (55.7)			
**Occupation Type** Support Workers	36 (30.8)	81 (70.5)	8.636	1	0.003*
Management & Professional	49 (50.5)	48 (49.5)			
**Work Place** Hospital	19 (36.5)	33 (63.5)	3.558	2	0.169
Health Office	32 (49.2)	33 (50.8)			
Clinic	34 (35.1)	63 (64.9)			
**Work Schedule** Shift	7 (31.8)	15 (68.2)	0.639	1	0.424
Office Hours	78 (40.6)	114 (59.4)			
**Smoking status** Non-smoker	78 (40.2)	116 (59.8)	0.205	1	0.651
Smoker / Ex-smoker	7 (35.0)	13 (65)			
**Knowledge** Good/Fair	27 (42.2)	37 (57.8)	0.232	1	0.630
Poor	58 (38.7)	92 (61.3)			

* Statistical significance at p-value < 0.05

Lastly, Table 6 highlights the significant associations between personal barriers, social barriers, and physical environment barriers with physical inactivity. Table 7 show the results of MLR analysis. Significant predictors of physical inactivity among pre-retirement HCWs included SPM/ STPM/ certificate education level (OR = 13.4, 95% CI: 2.47, 72.65, p-value = 0.003) and non-Malay ethnicity (OR = 4.7, 95% CI: 1.23, 18.38, p-value = 0.024). Besides that, personal barrier (OR = 1.56, 95% CI: 1.35, 1.79, p-value < 0.001), social barrier score (OR = 1.22, 95% CI: 1.06, 1.39, p-value = 0.004) and environment barrier score (OR = 1.47, 95% CI: 1.22, 1.77, p-value < 0.001) were also significant predictors of physical inactivity among pre-retirement HCWs.

**Table 6:** Association barriers against physical activity with physical inactivity (*n* = 214)

Factors	Unadjusted Coefficient	S.E.	Crude OR	95% CI for		*p*-value
				Lower	Upper	
**Barriers Against Physical Activity**						
Personal Factors	0.352	0.049	1.422	1.292	1.566	< 0.001*
Social Factors	0.093	0.035	1.098	1.024	1.176	0.008*
Physical Environment Factors	0.376	0.064	1.457	1.286	1.651	< 0.001*

* Statistical significance at p-value < 0.05, S.E. = Standard Error, OR = Odds Ratio, CI = Confidence Interval

**Table 7:** Multiple logistic regression (MLR) analysis between physical inactivity and its predictors among pre-retirement HCWs in Kuala Lumpur

Factors	Adjusted Coefficient	S.E.	Adjusted OR	95% CI for		*P* value
				Lower	Upper	
**Education Level**						
Post-Graduate/Degree			1			
Diploma	-0.486	0.532	0.627	0.221	1.778	0.380
SPM/STPM/Certificate	2.596	0.862	13.404	2.473	72.649	0.003*
**Ethnicity**						
Malay			1			
Non-Malay	1.558	0.691	4.747	1.226	18.381	0.024*
**Barriers Against Physical Activity**						
Personal Factors	0.442	0.073	1.556	1.350	1.794	< 0.001*
Social Factors	0.195	0.068	1.215	1.063	1.389	0.004*
Physical Environment	0.384	0.094	1.468	1.221	1.765	< 0.001*
Constant	-21.419	3.273				

*Notes* *Significance at p value < 0.05, S.E = standard error OR = odds ratio, CI = confidence

Variable selection method: Forward selection method

*no interaction *(*p* > 0.05),* no multicollinearity (VIF < 10)*,* no influential outlier*, Hosmer-Lemeshow goodness-of-fit test 0.526

Interval Nagelkerke R^2^ = 0.648, Cox and Snell R^2^ = 0.553, Nagelkerke R^2^ = 0.648

### The prediction of physical inactivity

The equation of probability for physical inactivity: 1/(1 + e^− z^).

Z = -21.419 + [-0.486 x Diploma] + [2.596 x SPM/STPM/Certificate] + [1.558 x Ethnicity] + [0.442 x personal Factors] + [0.195 x Social Factors] + [0.384 x Physical Environment].

## Discussion

In this study, the prevalence of physical inactivity among pre-retirement HCWs was 39.7%. It was higher than the prevalence among the general Malaysian population as reported in NHMS 2019 [11]. However, when compared with previous local studies among HCWs, the prevalence of physical inactivity among this group of professionals (32.8–45.6%) has always been higher than the general population [18–20]. Thus, our findings are consistent with the published prevalence of physical inactivity among HCWs. One recent study involving HCWs in a tertiary hospital reported a low physical inactivity prevalence of 2.0%. However, the study involved only nurses as compared to other studies that included HCWs of different occupations [28].

In terms of sociodemographic factors, there was a significant association between age with physical inactivity, similar to another study involving adults in Malaysia [29]. However, age was not a significant predictor following regression, likely because the age range of the population in this study was smaller than in other studies. However, non-Malay ethnic groups emerged as a significant predictor of physical inactivity, echoing another local study whereby the non-Malay ethnic groups in the general population reported 4.7 times higher odds of physical inactivity than Malays [30]. Cultural roles may play a role, for example, the interest of families often takes precedence over the individual’s interest in Indian culture, thus potentially resulting in less time for physical activity [31]. This is further supported by our findings in which the lack of free time for physical activity emerged as the highest agreeable item under social barrier factors.

Besides that, significant associations were also observed between income levels and physical inactivity. In this study, income levels were categorised as top 20%, middle 40%, and bottom 40% of socioeconomic status of populations in Kuala Lumpur according to the classification of the Department of Statistics Malaysia. This finding is consistent with another study involving HCWs in Kuala Lumpur [18]. The postulated reason could be persons with higher income levels are more engaged in administrative work with less physical activity performed during work. Meanwhile, there was also a significant association between education level and physical inactivity. Physical inactivity was more prevalent among pre-retirement HCWs with lower educational levels. The finding was in concordance with another study among HCWs in Putrajaya, Malaysia [20]. The findings can be explained by the fact that individuals with lower education levels are more likely to have a lower awareness of the importance of being physically active, thus predisposing them to physical inactivity [32].

Moreover, we also detected a significant association between occupational types and physical inactivity, with management and professional HCWs having a higher prevalence of physical inactivity than support staff. A study in Finland reported a similar association between different occupational types and physical inactivity [33]. However, our results contrasted another HCW study in Putrajaya, Malaysia whereby those in management and professional groups were more physically active compared to support staff [20]. The difference in findings could also be attributed to the population as this study recruited workers from various health facilities including hospitals and clinics whereas the study in Putrajaya involved only HCWs working in office-based settings. Support workers such as nurses, medical assistants, and healthcare assistants are involved in executing and implementing various services at the workplace, thus they are more likely to be involved in more physical activities at work compared to those under the management and professional group. However, this phenomenon needs to be explored as no published study available to support this finding.

In the social ecological model, personal characteristics play a crucial role in determining health behaviours, but a comprehensive understanding requires multi-dimensional approaches that take into account social and physical environments [34]. Thus, based on this model, healthy behaviours can be influenced by multiple factors. Some of these factors can pose as main barriers to healthy behaviours such as physical activity [34]. Furthermore, these barriers may present differently in different populations. Under the three domains of physical, social, and physical environment barriers, some factors emerged as significant predictors of physical inactivity. Based on the regression analysis, HCWs with one unit increase of personal, social, and physical environment barriers score recorded 1.6, 1.2, and 1.5 times higher odds of being physically inactive. The findings echoed another study involving HCWs in Putrajaya whereby predictors of physical inactivity included physical and social barriers alongside education level and smoking status [20].

Overall, personal-level barriers can present in multiple ways and may be perceived differently in different population settings. In a study involving adults in Singapore, the lack of time and tiredness were two of the commonest reported personal factors [35]. Firstly, social surroundings in the form of support for people to start or maintain healthy behaviours are vital to encourage the uptake of physical activity [36]. Evidence has shown that a good social network represents a form of positive support towards overcoming barriers and motivating individuals to become more physically active [37].

Meanwhile, physical environment factors related to healthy behaviour encompass the natural environment such as weather, and the built environment such as facility availability [36], both of which are barriers against the uptake of physical activity. The availability and accessibility of facilities are significantly associated with a higher physical activity level [38]. Therefore, it is important to review these factors in depth to implement the necessary strategies.

### Limitations and strength

This study has several limitations that need to be considered when interpreting the findings. As it involved only HCWs from a single health department, the findings may not be generalisable to other workplaces. Also, the self-administered online questionnaire can lead to inaccuracies in the collected data. Besides that, there could also be social desirability bias whereby respondents would answer the question to ensure that their answer is acceptable within the social norms instead of their true feelings or perspective. This is especially common if the study population involves HCWs who tend to provide more health-conscious answers in self-administered surveys like this. In addition, this study applied a cross-sectional design and thus causal relationship could not be concluded. This study also involved more females than males, potentially influencing the results as females may be less active compared to males. Furthermore, the distribution of ethnicities does not accurately represent the Malaysian population.

Despite these limitations, the study has notable strengths. The pre-retirement population between 45 and 60 years old has not been studied regularly in the past. Thus, the study findings shed new light on the issue of physical inactivity among this population. The outcomes can be used by other stakeholders who are undertaking similar efforts to high-risk groups with physical inactivity. Lastly, instead of a single-centre survey, this study involved staff working across multiple healthcare settings, i.e. hospitals, clinics, and health offices, hence providing more comprehensive input on the level of physical activity across different types of facilities.

### Recommendation

From the study findings, future interventions to mitigate physical inactivity among the working population can be customised accordingly. Intervention programmes must take into account high-risk population, especially those with lower education level and of non-Malay ethnicities. Besides improving the awareness and knowledge of HCWs via updated physical activity guidelines, they must also be educated about the importance of staying physically active. In addition, physical activity screening can be incorporated in workplace health screening programmes organised by the occupational health units. Following that, those with low physical activity levels can be identified and given suitable advice and techniques to increase their physical activity levels.

In addition, intervention programmes must also tackle the barriers to achieving sufficient levels of physical activity among the pre-retirement HCWs. Based on the study findings, physical environmental barriers, i.e. hot weather and rain as the main barriers reported by the HCWs. Therefore, indoor spaces for recreational physical activity such as mini gyms should be set up at the workplace to improve the uptake of physical activity among the workers. Besides that, indoor physical activity programmes should be organised regularly at the departmental or organisational levels to increase participation in physical activities.

Next, lack of spare time and self-discipline were other factors affecting the uptake of physical activity under the social and personal barriers. With these factors in mind, committees should be set up with the help of the occupational and safety health units as well as health education units in each health facility to organise physical activities that workers can join as group activities. Regular group sessions for recreational physical activities for 20–30 min daily can help to overcome self-discipline and time management issues among HCWs. In addition, AI-driven applications are recommended to promote active living through personalized activity plans, fitness tracking, and health incentives. This approach can encourage consistent physical activity across different groups and help ensure a more diverse ethnic representation.

## Conclusion

This study shows a worrying prevalence of physical inactivity among pre-retirement healthcare workers that is even higher than the general population in Malaysia. The findings highlight the importance of focusing the preventive strategies among non-Malay workers and those with lower education levels. It is also vital to address all the physical, social, and environmental barriers towards physical inactivity. By prioritising these factors, employers and stakeholders will be able to establish better workplace health promotion and address the issue of physical inactivity more efficiently.

### Data management

Online questionnaires were used for anonymised data collection. Study data and personal information were extracted using Microsoft excel and stored under the records and documentation system of the Universiti Putra Malaysia’s system. All data will be destroyed five years later. Any study participant who requests for the study findings will be informed via emails.

## Data Availability

All relevant data are within the manuscript.
